# Signalling through MyD88 drives surface expression of the mycobacterial receptors MCL (Clecsf8, Clec4d) and Mincle (Clec4e) following microbial stimulation

**DOI:** 10.1016/j.micinf.2016.03.007

**Published:** 2016

**Authors:** Bernhard Kerscher, Ivy M. Dambuza, Maria Christofi, Delyth M. Reid, Sho Yamasaki, Janet A. Willment, Gordon D. Brown

**Affiliations:** a Institute of Medical Sciences, University of Aberdeen, Foresterhill, Aberdeen AB25 2ZD, UK; b Division of Molecular Immunology, Medical Institute of Bioregulation, Kyushu University, Japan

**Keywords:** C-type lectin receptor, Clec4d, Dectin-3, Clec4e, MyD88, TLR signalling

## Abstract

The heterodimeric mycobacterial receptors, macrophage C-type lectin (MCL) and macrophage inducible C-type lectin (Mincle), are upregulated at the cell surface following microbial challenge, but the mechanisms underlying this response are unclear. Here we report that microbial stimulation triggers Mincle expression through the myeloid differentiation primary response gene 88 (MyD88) pathway; a process that does not require MCL. Conversely, we show that MCL is constitutively expressed but retained intracellularly until Mincle is induced, whereupon the receptors form heterodimers which are translocated to the cell surface. Thus this “two-step” model for induction of these key receptors provides new insights into the underlying mechanisms of anti-mycobacterial immunity.

## 1 Introduction

Tuberculosis (TB), caused by *Mycobacterium tuberculosis*, is one of the most prevalent infectious diseases with an estimated two billion individuals infected worldwide [1]. Interestingly, only a fraction of these carriers develop active disease, a process that is not yet fully understood [2]. Human genetic association studies have demonstrated an important role for innate immune receptors and their signalling pathways in TB susceptibility and disease progression [1]. Pattern recognition receptors on innate immune cells play a crucial role in both homoeostasis and host defence against pathogens. Toll-like receptors and C-type lectin receptors (CTLRs) are the major families of surface expressed PRRs that detect pathogen associated molecular patterns (PAMPs), triggering complex signalling cascades to initiate host defences such as the release of cytokines and chemokines, which are key for the activation and recruitment of leukocytes during TB [1], [3], [4]. Toll-like receptors (TLRs) directly sense a variety of mycobacterial components such as glycolipids and glycoproteins (TLR2/1), diacylated lipoproteins (TLR2/6), heat shock protein 60/65 (TLR4), and DNA motifs (TLR9) [1], [5], [6]. More recently, several members of the Dectin-2 family of CTLRs were shown to play a role in anti-mycobacterial immunity through recognition of trehalose dimycolate (MCL, Mincle) or mannose-capped lipoarabinomannan (Dectin-2) [7], [8], [9], [10]. These receptors associate with the signalling adaptor FcRγ for surface expression and the transduction of an activating signal [11]. Of particular importance here is MCL, which was shown to play a non-redundant role in a murine pulmonary TB model [10]. The current model for mycobacterial trehalose dimycolate (TDM) recognition by MCL and Mincle suggests a constitutive expression of MCL, which recognises the lipid moiety of TDM [12]. This induces an activation signal, transduced through the CARD9/Bcl10/MALT1 axis, leading to NF-κB p65 initiation of Mincle expression [13], [14].

Although MCL was described as a constitutively expressed receptor and is highly expressed on resident peritoneal macrophages, we and others have recently demonstrated that its surface expression on myeloid cells can be significantly upregulated by microbial stimuli *in vitro* and pulmonary infection with *Mycobacterium bovis* Bacillus Calmette-Guérin (BCG) *in vivo* [15], [16]. MCL and Mincle were reported to form functional heterodimers [15], [16]. Notably, each receptor was essential for surface expression of its heterodimeric partner under naive conditions as well as during upregulation of expression on BCG-infected bone marrow-derived macrophages (BMM) [15]. In this study, we investigated the mechanism underlying the induction of MCL and Mincle surface expression following microbial stimulation.

## 2 Materials and methods

### 2.1 Cells, bacterial strains, mice and reagents

C57BL/6 wildtype and matching Mincle^−/−^, MCL^−/−^ and MyD88^−/−^ mice (on a C57BL/6 background) were housed with access to water and food *ad libitum* in the specific pathogen free animal facilities at the University of Aberdeen (UK). Procedures were carried out in accordance with approved protocols from the UK Home Office under project licences 60/4007 and 70/8073. Bone marrow-derived macrophages (BMM) were generated in the presence of conditioned L929 supernatant in complete RPMI medium (Gibco), as described previously [17]. *M. bovis* BCG strain Pasteur was grown on Middlebrook 7H10 agar or in Middlebrook 7H9 broth (BD) [10]. BMM were plated at 2.5 × 10^5^ cells/well in 24 well plates (Thermo Scientific) for flow cytometry, or 1 × 10^6^ cells/well in 6 well plates (Sigma) for total protein. Stimulations with BCG (multiplicity of infection: 1), TLR-4 agonist LPS (100 ng ml^−1^, Sigma) and TLR-2/1 agonist Pam_3_CSK_4_ (100 ng ml^−1^, Invivogen) were carried out as described previously [15], [18].

### 2.2 Flow cytometry

For analysis of receptor expression, cells were stained and analysed by flow cytometry as described previously [15], in the Iain Fraser Cytometry Centre at the University of Aberdeen. Briefly, cells were harvested, passed through a 40 μm cell strainer and red blood cells lysed in PharmLyse (BD), before staining in FACS block (PBS, 0.5% BSA, 5% rabbit serum, 5 mM EDTA, 1 mM azide) containing 4 μg/ml 2.4G2 Fc-receptor block. For intracellular staining, cells were fixed in 1% paraformaldehyde in PBS, followed by permeabilisation in 0.05% saponin (Sigma) in FACS Block. Antibodies used were CD45 (clone 104), CD11b (clone M1/70), F4/80 (clone Cl:A3-1), MCL-biotin (clone 3A4 [15]), Mincle-biotin (clone 4A9 [7]) and Dectin-2-biotin (clone 11E4 [19]). Biotinylated antibodies were detected with an appropriate streptavidin conjugate (BD, Invitrogen) and measured by flow cytometry on LSR Fortessa or Array instruments (BD) and data analysed using FlowJo v.10.0.8. Mean fluorescent intensity (MFI) data reported are MFI of anti-CTLR minus MFI of isotype control.

### 2.3 Western blot

Cells were plated and stimulated as indicated in the figures followed by lysis in ice cold RIPA buffer (50 mM Tris pH 8, 150 mM NaCl, 1% NP-40, 0.5% sodium deoxycholate, 0.1% SDS, 1 mM EDTA) containing complete EDTA-free protease inhibitor cocktail (Roche). Western blots on equal amounts of protein quantitated by BCA assay (Pierce) were performed following conventional protocols using the NuPAGE system (Invitrogen) and probed with antibodies as indicated in the figures. Equal loading was demonstrated by stripping the blots (re-blot mild buffer, Millipore) and re-probing the membranes with mouse anti-mGAPDH (clone mAbcam 9484).

### 2.4 Data analysis

Data was compiled and analysed using FlowJo v10.0.8, Excel and Graphpad Prism v5.04, and analysed with ANOVA and Bonferroni post-test. Data was considered statistically significant if *p* < 0.05.

## 3 Results

### 3.1 Surface expression of MCL and Mincle is induced by TLR ligands

**Fig. 1:**
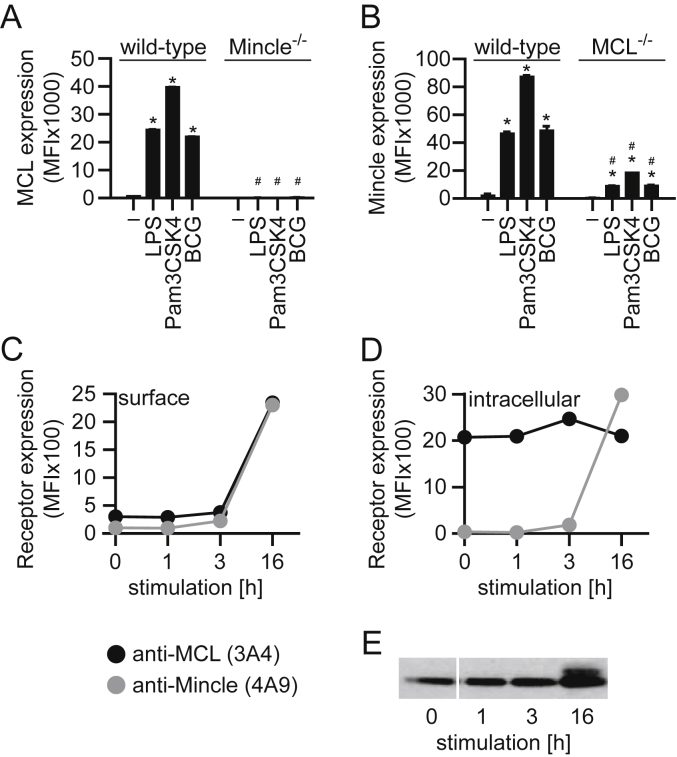
Microbial stimuli promote translocation of Mincle and MCL to the cell surface. BMM were treated for 16 h (h) with various microbial stimuli, as indicated, and surface (A–C) and intracellular (D) protein expression assessed for MCL or Mincle, by flow cytometry as indicated. MCL total cellular protein was also analysed over time by Western blot analysis (E). Data shown from individual experiments in duplicate and are representative of two independent experiments. Data shown as mean + standard deviation (SD). *, *p* < 0.05 compared to untreated control of the same strain. #, *p* < 0.05 compared to the same treatment of the wild-type strain. –, unstimulated control.

We recently reported that microbial challenge led to increased expression of MCL and Mincle in an inter-dependent fashion [15]. To confirm these findings, we stimulated wild-type, Mincle^−/−^ and MCL^−/−^ BMM with LPS, Pam_3_CSK_4_ or *M. bovis* BCG and assessed receptor surface expression by flow cytometry. Consistent with our previous observations, stimulation with microbial agonists strongly increased surface expression of both MCL (Fig. 1A) and Mincle (Fig. 1B) on wild-type cells [15]. Furthermore, the induced surface expression of each of these CTLRs was dependent on co-expression of its heterodimeric partner, since expression of MCL or Mincle was substantially reduced on Mincle^−/−^ (Fig. 1A) or MCL^−/−^ cells (Fig. 1B), respectively. Notably, MCL expression was completely absent on Mincle^−/−^ cells, but Mincle could still be induced on MCL^−/−^ cells, albeit at low levels. This suggests that expression of Mincle is not absolutely dependent on MCL, as we had previously observed in alveolar macrophages [15].

### 3.2 MCL is constitutively expressed but retained intracellularly

While Mincle expression has been suggested to be controlled by MCL on a transcriptional level, MCL is thought to be a constitutively expressed receptor, at least based on analysis of mRNA expression [7], [13]. To gain further insight into the underlying mechanism of induced surface expression, we analysed the temporal dynamics of MCL and Mincle protein expression in BMM following BCG stimulation by flow cytometry. BCG was chosen for this analysis as it is a physiologically relevant complex microbial ligand, compared to a selected TLR agonist. Both receptors remained absent from the cell surface for the first hour post stimulation and demonstrated only a minor increase after 3 h before significant surface expression was detected at 16 h post stimulation (Fig. 1C). There was no change in receptor expression in the absence of stimulation (data not shown). Surprisingly, we did not observe surface expression of MCL preceding that of Mincle following stimulation, at least at the time points analysed. Next, we investigated the possibility of an intracellular pool of MCL, consistent with its reported constitutive mRNA expression profile. Therefore we performed total protein staining on fixed and permeabilised cells during a time course experiment, as above. Indeed, we detected substantial amounts of intracellular MCL protein in naïve BMM, with total protein levels remaining largely unchanged following microbial stimulation, as determined by flow cytometry (Fig. 1D). In contrast, the expression of intracellular Mincle, mirrored the time course of the surface expressed protein (Fig. 1D). The presence of a total MCL protein pool over the entire time frame of this analysis was confirmed by Western blot on whole cell lysates, although there appeared to be a slight increase in MCL levels at 16 h (Fig. 1E). Thus, MCL is a constitutively expressed, but intracellularly retained receptor, whose surface expression requires co-expression of Mincle, which is itself only induced following an activation signal.

### 3.3 MyD88 is essential for the induction of MCL surface expression following microbial stimulation

**Fig. 2:**
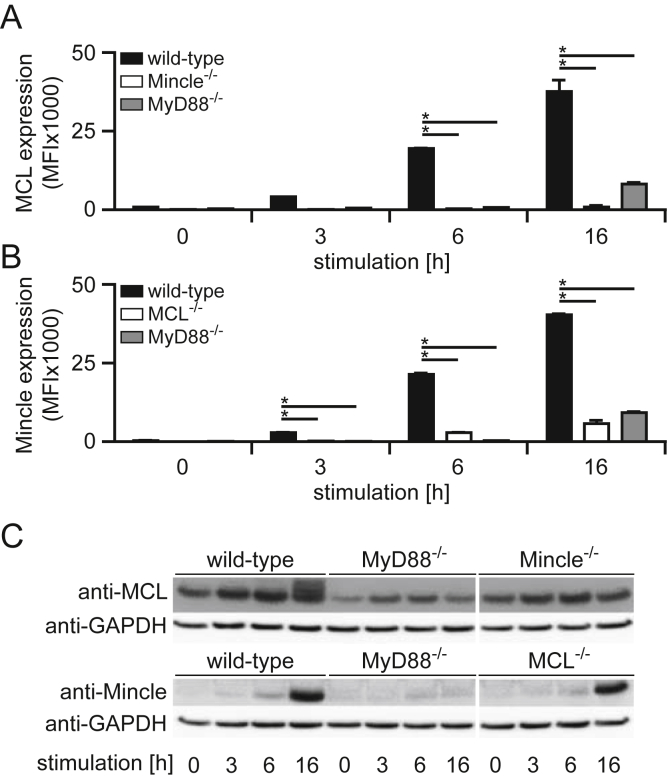
MyD88 is required for MCL translocation the cell surface of BMM following BCG challenge. BMM from various mouse strains, as indicated, were stimulated with BCG for the specified number of hours and surface expression of MCL (A) or Mincle (B) analysed by flow cytometry. (C) Total protein levels of the receptors in BMM cell lysates, following BCG stimulation, were analysed by Western blot. The data show mean + SD of duplicates and are representative of at least 2 independent experiments. *, *p* < 0.05 compared to wild-type.

Since TLR agonists induce MCL and Mincle surface expression (Fig. 1A and [15]), we hypothesised that TLR-signalling may play a role in this process. Further support for this hypothesis stems from published microarray databases, where upregulation of MCL and Mincle during pulmonary *Chlamydia* infection was shown to be MyD88-dependent [20]. We therefore stimulated BMM from wild-type, MCL^−/−^, Mincle^−/−^ and MyD88^−/−^ mice and analysed receptor surface expression by flow cytometry (Fig. 2A and B). As we had observed previously, MCL expression following BCG challenge increased over time in wild-type mice, and required the presence of Mincle (Fig. 2A). In fact, in these experiments we could detect increased surface expression of these receptors by 6 h after stimulation. Notably, loss of MyD88 led to substantial reductions in surface expression of MCL, demonstrating the importance of this TLR signalling pathway in this process. Similarly, the surface expression of Mincle mirrored that of MCL and also required MyD88 (Fig. 2B). Analysis of whole cell lysates, revealed the presence of MCL in Mincle and MyD88-deficient cells (Fig. 2C), consistent with a constitutive expression profile [7]. In contrast, Mincle was induced following stimulation and its levels increased over time, even in the absence of MCL (Fig. 2C). However, this induction did not occur in MyD88^−/−^ cells, revealing a critical role for this signalling pathway in induction of Mincle and the subsequent surface expression of both Mincle and MCL.

### 3.4 MyD88 is dispensable for constitutive expression of MCL and Mincle

**Fig. 3:**
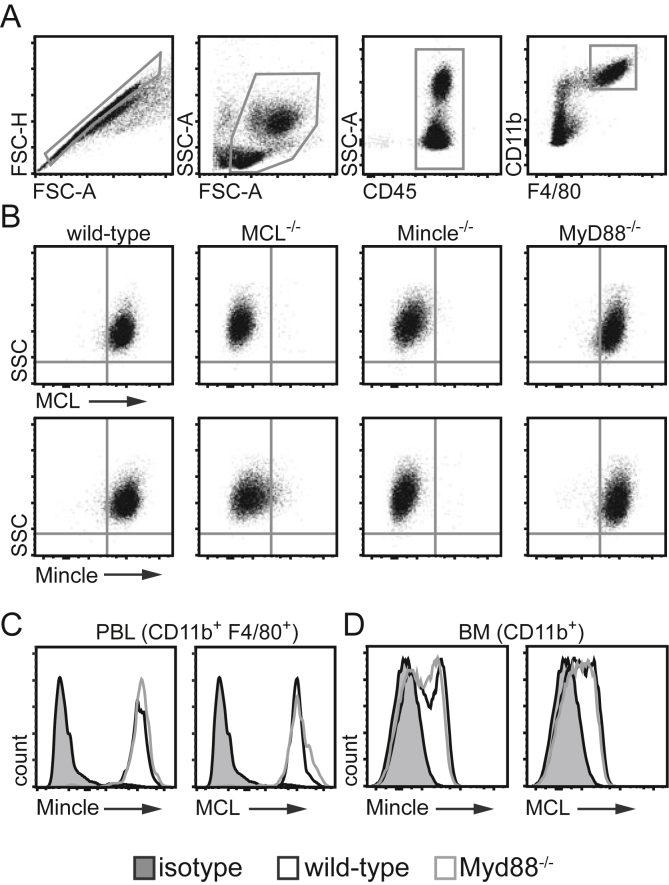
MyD88 is dispensable for basal MCL/Mincle expression on resident myeloid cells. (A) Example gating strategy for myeloid cells (here resident peritoneal macrophages) for flow cytometric analysis of receptor expression on cells from naïve mice. MCL and Mincle expression on (B) resident peritoneal macrophages, (C) CD11b^+^F4/80^+^ monocytes in peripheral blood (PBL) and (D) CD11b^+^ cells in bone marrow (BM). Data is representative of at least 3 mice from two independent experiments.

MCL and Mincle are highly expressed on the surface of naïve resident peritoneal macrophages and peripheral blood leukocytes (PBL), in an interdependent manner [15]. Thus we next explored whether MyD88 signalling is required for surface expression of these CTLRs in naïve mice. Resident peritoneal macrophages have the highest levels of surface receptor expression [15], [21] and flow cytometric analysis of CD11b^+^F4/80^+^ resident macrophages (Fig. 3A) confirmed that high levels of MCL and Mincle are expressed at the surface of these cells in an interdependent fashion (Fig. 3B). Notably, surface expression of both receptors was not affected by the absence of MyD88 (Fig. 3B). These findings were supported by similar receptor expression profiles on PBL (Fig. 3C) and bone marrow cells (Fig. 3D). Therefore, canonical TLR signalling through MyD88 is not required for surface expression on the naïve myeloid cells examined here, such as resident peritoneal macrophages.

## 4 Discussion

Our understanding of the role of members of the Dectin-2 family of C-type lectin receptors in anti-mycobacterial immunity has advanced significantly over the last few years. Notably, Dectin-2, Mincle and MCL have all been shown to recognise mycobacterial ligands with MCL, in particular, demonstrated to play a key role in both mouse and human [10]. Recently, MCL was reported by several groups, including ours, to form a functional heterodimer with Mincle [15], [16], [22]. Murine MCL co-immunoprecipitates with Mincle and FcRγ and the levels of MCL surface expression correlates with those of Mincle in both primary and transduced cells [15], [16]. Consistent with previous reports [13], [15], we show here that following stimulation with microbial components the high level of Mincle expression at the cell surface is dependent on MCL, and vice versa. While MCL appears to increase Mincle surface expression when overexpressed *in vitro*, MCL has also been suggested to play a critical role in regulating Mincle expression at a transcriptional level [13], [16].

Our data support a model of constitutive expression of MCL and induction of Mincle following microbial stimulation. However, we demonstrate that the induction of Mincle at the protein level does not require MCL. Rather, we have found that induction of Mincle following microbial stimulation requires the MyD88 pathway, suggesting possible involvement of TLR(s) in this process. Various TLRs have been implicated in anti-mycobacterial immunity, but TLR2/4/9 triple-deficient mice are able to control TB infection [23]. In contrast, MyD88^−/−^ mice rapidly succumbed to the disease [24], a phenotype linked to defects in IL-1 signalling [25]. Indeed, preliminary analysis of TLR4 deficient BMM, revealed little defect in surface expression of MCL or Mincle following BCG stimulation (data not shown).

In conclusion, we propose that the MyD88 pathway, rather than MCL signalling, is key for Mincle expression following microbial challenge. Our data suggests a “two-step” model for surface expression of MCL and Mincle following microbial challenge in bone-marrow macrophages. In this model, microbial stimulation induces MyD88-mediated signalling resulting in the upregulation of intracellular levels of Mincle protein, that are detectible within 6 h of stimulation. Mincle then forms heterodimers with constitutively expressed MCL, resulting in translocation of both CTLRs to the cell surface where they mediate their anti-microbial activities. Our previous *in vivo* observations support this model: high level of expression of these receptors was only induced in the lungs of mice following mycobacterial infection [15]. Why such an important innate recognition system [10] is not constitutively expressed at high levels is still unclear. While clearly sufficient to induce protective anti-mycobacterial responses [10], the low levels of receptor expression in naïve animals may help prevent unwanted inflammatory responses to the endogenous ligands that are also recognised by these CTLRs [11]. Future work will be directed at understanding the underlying mechanisms of surface expression of Mincle/MCL in resident macrophage populations in naïve animals, where MyD88 is not required, and how these regulatory mechanisms are coordinated *in vivo* during mycobacterial infection.

## Conflict of interest

The authors have no conflict of interest to declare.
